# Automated Detection of Soma Location and Morphology in Neuronal Network Cultures

**DOI:** 10.1371/journal.pone.0121886

**Published:** 2015-04-08

**Authors:** Burcin Ozcan, Pooran Negi, Fernanda Laezza, Manos Papadakis, Demetrio Labate

**Affiliations:** 1 Dept. of Mathematics, University of Houston, Houston, Texas, United States of America; 2 Dept. of Pharmacology and Toxicology, University of Texas Medical Branch, Galveston, Texas, United States of America; Tufts University, UNITED STATES

## Abstract

Automated identification of the primary components of a neuron and extraction of its sub-cellular features are essential steps in many quantitative studies of neuronal networks. The focus of this paper is the development of an algorithm for the automated detection of the location and morphology of somas in confocal images of neuronal network cultures. This problem is motivated by applications in high-content screenings (HCS), where the extraction of multiple morphological features of neurons on large data sets is required. Existing algorithms are not very efficient when applied to the analysis of confocal image stacks of neuronal cultures. In addition to the usual difficulties associated with the processing of fluorescent images, these types of stacks contain a small number of images so that only a small number of pixels are available along the z-direction and it is challenging to apply conventional 3D filters. The algorithm we present in this paper applies a number of innovative ideas from the theory of directional multiscale representations and involves the following steps: (i) image segmentation based on support vector machines with specially designed multiscale filters; (ii) soma extraction and separation of contiguous somas, using a combination of level set method and directional multiscale filters. We also present an approach to extract the soma’s surface morphology using the 3D shearlet transform. Extensive numerical experiments show that our algorithms are computationally efficient and highly accurate in segmenting the somas and separating contiguous ones. The algorithms presented in this paper will facilitate the development of a high-throughput quantitative platform for the study of neuronal networks for HCS applications.

## Introduction

The ability of neurons to form mature synapses and functional connections in culture is a fundamental property that has allowed the first milestone studies in molecular neuroscience. In recent years, improved neuronal culturing techniques combined with sophisticated fluorescence microscopy have significantly expanded the initial scope of *in vitro* studies. They are now instrumental tools for large-scale studies of cultured neuronal networks used to identify phenotypic changes induced by chemical or biological agents in the context of brain disease models. Yet, such revolution in the field has not been paralleled by adequate quantitative methods for neuronal feature detection, extraction and analysis, limiting the potential throughput of *in vitro* models.

Automated identification of the primary components of a neuron and extraction of its sub-cellular features are essential steps in such quantitative studies. High-content screenings (HCS), for instance, require the identification and extraction of multiple morphological features of neurons, such as soma shape and volume, neurite length and branching properties. Such complex information, usually compiled from multi-channel fluorescence images, requires automated processing methods to handle large batches of data and establish a confident statistical basis for a reliable prediction model. With the rapid and widespread rise in the use of HCS in basic science settings, automated detection of cell compartments from fluorescent images is an area of very active research [1–4].

In this paper, we concentrate on the automated detection of soma location and surface morphology in fluorescent images of cultured neurons, a challenging problem that has special importance for several reasons. In particular, *in vitro* phenotypic screenings of large scale neuronal cultures for drug-discovery and biomarker identification frequently require to quantify spatial distribution and expression levels of analytes of interest inside the soma [5, 6]. In addition, the detection of soma locations is a critical step to compute the centerline trace and extract the graph representation of each individual neuron, since the location of the soma is the main vertex of such a graph [7, 8]. Furthermore, accurate extraction of soma’s surface morphology is one of the most important characteristics for discriminating different types of neurons [9].

Of all sub-cellular components of a neuron, somas are among the most challenging to detect automatically in fluorescence images, due to the lack of robust markers and the uneven distribution of fluorescence signals. Automated soma/cell detection methods in many image analysis packages rely on contrast enhancement and image intensity thresholding [10, 11] and attempt the identification of somas by generating binary masks. These methods can be quite effective in phase-contrast microscopy [11, 12], but the extension of these methods to images captured by other types of microscopy is difficult and not always very effective. In fluorescence imaging, in particular, the image contrast is typically much lower and high fluorescent intensity values can be found outside the soma region. In fluorescent image of neuronal cultures, somas are usually visible with the aid of the Microtubules Associated Protein 2 (MAP2) antibody staining or nuclei markers (such as DAPI or TROPO-3), neither of which are soma-selective. The MAP2 protein is diffusely distributed in the somato-dendritic compartment and, as a result, MAP2 masks include both somas and dendrites. Nuclear staining, on the other hand, targets only nuclear DNA and excludes the cytoplasmic region of the soma surrounding the nucleus.

To address this problem, a number of methods have been proposed to process images in which somas and other structures exist in the same fluorescence channel. Some of these methods use combinations of smoothing and morphological operators [7, 13, 14] or specialized filters such as the Laplacian-of-Gaussian (LOG) [15] and can be rather effective to detect somas in 2D. However these types of methods tend to be rather inaccurate since they are very sensitive to the irregularities of the fluorescence signal and have proven to be either impractical or inefficient to extend to 3D. In particular, the use of LOG to detect local maxima of the fluorescence intensity signal can lead to a high degree of false positives due to the irregular intensity profiles, causing detection of more than one soma candidate within a given neuron. To address these limitations, several ideas have been proposed, such as the detection of the soma area in the three 2D orthogonal projection images of the original 3D image stack using 2D morphological closing [8]. Of special note is the algorithm recently proposed by [16] that, in addition to combining smoothing, morphological operators and adaptive thresholding to detect the soma volume from the 2D orthogonal projection images, applies an ingenious variant of the Rayburst sampling method [17] to capture the surface of the soma.

None of those methods, though, are directly applicable to the type of imaging data which are the preferred platform for HCS. Confocal images of neuronal cultures used for HCS typically consist of stacks containing 15–30 images, so that the ‘data cube’ is very thin along one of its axes: only 15–30 pixels are available along the *z*-direction, as compared with the *x* and *y* directions where the typical length can be 512 pixels or more. As a result, it is inefficient or even impossible to process these data as true volumes using conventional 3D filters or adapting some of the ideas mentioned above. For example, the 2D orthogonal projections into the planes containing the *z* axis would not be very informative for the detection of the soma volume, due to the lack of a sizeable *z* dimension. An additional challenge is that, due to the acquisition process, in cultured neuronal images the contrast changes significantly along the stack and reduces very rapidly on the optical slices that are farther away from the light source.

To address these challenges, in this paper we introduce an innovative method for the automated detection and accurate segmentation of somas from high-resolution confocal images of cultured neurons. Our approach includes two separate algorithms for the 2D and 3D analysis. Due to the difficulty in processing confocal imaging data as true volumes, it is customary to convert the MAP2-stained confocal image stacks into 2D images by projecting the stacks along the *z*-axis. Consistently, our first soma detection algorithm is designed to deal with these types of 2D images. Our approach includes an SVM-based segmentation routine to separate the neurons from the background, followed by a dedicated routine to separate the somas from the neurites. The latter routine combines innovative ideas from the theory of directional multiscale representations (developed by some of the authors) together with variational methods. This approach enables very accurate detection of the somas in 2D, including the separation of clustered or contiguous somas. Our second algorithm is designed to extract the volumetric information of somas from the 3D image stacks and relies on our 2D soma detection algorithm to de-block the image stacks into subvolumes containing a single soma. Within each block, we apply a specialized surface detection routine, based again on our multiscale-analysis framework, to efficiently extract the surface morphology of the soma.

## Materials and Methods

### Specimen preparation and imaging

The image datasets used in the present work are primary hippocampal neuronal cultures that were prepared in Dr. Laezza’s Laboratory at the Department of Pharmacology & Toxicology of the University of Texas Medical Branch.

Banker’s style hippocampal neuron cultures were prepared from embryonic day 18 (E18) rat embryos as described in previous work [5]. Briefly, following trituration through a Pasteur pipette, neurons were plated at low density (105 × 105 cells/dish) on poly-L-lysine-coated coverslips in 60 mm culture dishes in MEM supplemented with 10% horse serum. After 24 h, coverslips (containing neurons) were inverted and placed over a glial feeder layer in serum-free MEM with 0.1% ovalbumin and 1 mM pyruvate (N2.1 media; Invitrogen, Carlsbad, CA) separated by approx. 1 mm wax dot spacers. To prevent the overgrowth of the glia, cultures were treated with cytosine arabinoside at day 3 in vitro (DIV).

Hippocampal neurons (DIV14) were fixed in fresh 4% paraformaldehyde and 4% sucrose in phosphate-buffered saline (PBS) for 15 min. Following permeabilization with 0.25% Triton X-100 and blocking with 10% BSA for 30 min at 37°C, neurons were incubated overnight at room temperature with the following primary antibodies: mouse anti-FGF14 (monoclonal 1:100; Sigma Aldrich, St Louis, MO), rabbit anti-PanNav (1:100; Sigma, St Louis, MO) and chicken anti-MAP2 (polyclonal 1:25000; Covance, Princeton, NJ) diluted in PBS containing 3% BSA. Neurons were then washed 3 times in PBS and incubated for 45 min at 37°C with appropriate secondary antibodies as described for brain tissue staining. Coverslips were then washed 6 times with PBS and mounted on glass slides with Prolong Gold anti-fade reagent.

Confocal images were acquired with a Zeiss LSM-510 Meta confocal microscope with a 63X oil immersion objective (1.4 NA). Multi-track acquisition was done with excitation lines at 488 nm for Alexa 488, 543 nm for Alexa 568 and 633 nm for Alexa 647. Respective emission filters were band-pass 505–530 nm, band-pass 560–615 nm and low-pass 650. Z-stacks were collected at *z*-steps of 1 *μ*m with a frame size of 512 × 512, pixel time of 2.51 *μ*s, pixel size 0.28 × 0.28 *μ*m and a 4-frame Kallman averaging. Acquisition parameters, including photomultiplier gain and offset, were kept constant throughout each set of experiments.

All the animal procedures were performed in accordance to the University of Texas Medical Branch at Galveston, Institutional Animal Care and Use Committee(IACUC) approved protocols. The University of Texas Medical Branch at Galveston operates in compliance with the United States Department of Agriculture Animal Welfare Act, the Guide for the Care and Use of Laboratory Animals, and IACUC approved protocols.

### Shearlet transform

We recall some background on the shearlet transform. This method will play a major role in our algorithm.

The shearlet framework is one of the most prominent methods to have emerged in the area of multiscale analysis in recent years [18]. It was introduced to overcome the limitations of conventional wavelets in dealing with multidimensional data, and it offers the advantage of combining multiresolution analysis of classical wavelets with high directional sensitivity, so that it is especially efficient at handling images containing edges and surface boundaries. In fact, the analyzing atoms of the shearlet system form a collection of waveforms {*ψ*_*a*,*s*,*p*_} defined not only over a range of *scales* and *locations*, like wavelets, but also over a range of *orientations*. As a result, the *shearlet transform*, defined by projecting an image *f* into the set of analyzing shearlets, maps the input image *f* into a transformed image

$$\begin{array}{c} f⟶𝒮f\left(a,s,p\right)=\left\langle f,\psi_{a,s,p}\right\rangle , \end{array}$$

where the values 𝒮*f*(*a*, *s*, *p*) depend on a scale variable *a* > 0, a shear variable *s* ∈ ℝ, controlling orientations, and a location variable *p* ∈ ℝ^*n*^, where *n* = 2 for a planar image or *n* = 3 for a 3D-image (e.g., an image stack). In a nutshell, the elements 𝒮*f*(*a*, *s*, *p*) encode the information of the image *f* in a localized elongated window centered at *p* whose orientation is controlled by *s* and whose size is controlled by *a*, with the window becoming increasingly thinner at finer scales, i.e., as *a* becomes smaller.

The most remarkable property of the shearlet transform is that it provides a precise analytical and computational tool to detect edges and surface boundaries of an image *f*. In particular, *if *f* is an image containing an edge, then the shearlet transform 𝒮*f*(*a*,*s*,*p*) exhibits rapid decay, as *a* becomes smaller, everywhere except for those points *p* located on the edge and those *s* corresponding to the normal orientation to the edge at *p*,* with a similar property holding for a 3D-image [19–21]. This implies that one can detect edges and surfaces in 2D and 3D images, respectively, by computing the shearlet transform. This method was successfully implemented and illustrated by one of the authors and other collaborators [22, 23]. More generally, one can use the shearlet transform to study local directional patterns in images. This idea plays a major role in the construction of our soma detection algorithm.

### 2D soma detection algorithm

Our algorithm for 2D soma detection is applied on 2D images obtained by projecting a confocal image stack (comprising typically about 15–30 optical sections) along the axis perpendicular to the image plane (the *z* axis). This 3D-to-2D conversion is common in manual or semi-manual analysis of neuronal cultures. The most common projections are the *average intensity projection* (AIP) that outputs an image, wherein each pixel stores the average intensity over all images in stack at corresponding pixel location, and the *maximum intensity projection* (MIP), that creates an output image where each of the pixels contains the maximum value over all images in the stack at the particular pixel location.

**Fig 1:**
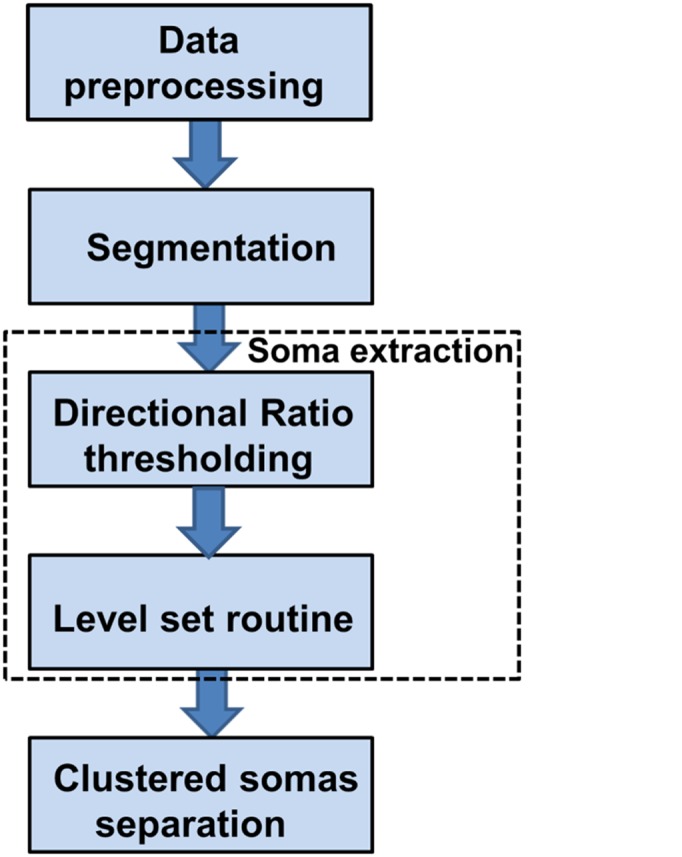
Proposed algorithm for 2D soma detection.

Our algorithm follows the procedure shown in Fig. 1 and consists of the following steps: (1) a preprocessing routine to denoise the image; (2) a segmentation routine to separate the neurons from the background and to prepare the data for the next processing steps; (3) a routine that extracts the somas from the segmented images and includes Directional Ratio and level set routines; (4) a routine to separate those somas that are clustered together. As we will describe below, each step of the algorithm is completely automatic and does not require manual intervention.

#### Image preprocessing

The design of a highly efficient data preprocessing module is essential to take full advantage of the capabilities of instrumentation and enable accurate and robust processing of the acquired data. Our preprocessing algorithm addresses the major source of degradation of confocal images: the noise introduced by the random nature of the photon-counting process at the detector, which can be modeled as a Poisson-distributed random process. To remove the noise, we apply the *shearlet shrinkage* denoising algorithm from [24, 25]. This algorithm is a refinement of the celebrated *wavelet shrinkage* denoising method and consists of the following steps. First the image is transformed using the continuous shearlet transform. Next the shearlet transform coefficients are filtered using an appropriate shrinkage function whose parameters are automatically determined from the data, based on the estimate of the noise level [24]. Finally the denoised image is obtained by computing the inverse shearlet transform. The excellent denoising performance of this algorithm takes advantage of the sparsity properties of shearlets, that have optimally sparse representation properties for a large class of images [18, 26]. Thanks to these sparsity properties, this method is particularly efficient at removing noise without blurring edges [24, 27]. Overall, the boundaries of the structures in an image look sharper after the application of this denoising method.

#### Segmentation

Segmentation has to goal to separate the neurons from the background. For this task, we adopted an algorithm recently developed by some of the authors that is based on support vector machines (SVMs) and whose main novelty is the use of features generated by a set of multiscale isotropic Laplacian filters acting as self-steerable filters for a quick and efficient binarization of the axonal and dendritic structure [28–30]. As for many algorithms of this type, the proper classification stage of the algorithm is preceded by a training stage of the classifier. This is the most computationally-intensive part of the algorithm but need to be run only once as long as the segmentation algorithm is applied to images of the same type (e.g., same cell type and microscope setting). The whole procedure is fully automated. We refer the reader to the references cited above for more details about the algorithm.

#### Soma extraction

Our method to identify the soma within the segmented neuron applies an innovative idea that takes advantage of the directional sensitivity of the shearlet transform in order to separate isotropic blob-like regions from anisotropic vessel-like structures. The mathematical framework for this method was developed by the authors in [31, 32], where we introduced the following measure of directional coherence. We define the *Directional Ratio* of an image *f* at scale *a* > 0 and location *p* ∈ ℝ^2^ as the quantity

$$\begin{array}{c} {𝒟}_{a}f\left(p\right)=\frac{\inf_{s}\left\{|𝒮f\left(a,s,p\right)|\right\}}{\sup_{s}\left\{|𝒮f\left(a,s,p\right)|\right\}} \end{array}$$

where 𝒮*f*(*a*, *s*, *p*) is the shearlet transform of *f* defined above. This quantity, that ranges between 0 and 1, measures the strength of the directional coherence of *f* at scale *a* and location *p*. In [31], we proved that if *f* is a *cartoon-like neuron*, that is, an idealized model image containing a union of blob-like and vessel-like structures, then, when *a* is sufficiently small, there exists a threshold which is significantly less than 1 such that the Directional Ratio doesn’t exceed that threshold when *p* is located inside the vessel-like structure. On the other hand, the Directional Ratio varies wildly in more isotropic (e.g., blob-like) structures. This suggests that we can identify the somas with respect to the neurites, if we compute the Directional Ratio inside the already segmented neuronal structure at an appropriate scale and set a small threshold. The existence of such a threshold even in the discrete setting was verified numerically in [31].

For the discrete implementation of the Directional Ratio, in this paper, we will use a discrete version of the shearlet transform defined in Section 1, of the form

$$\begin{array}{c} 𝒮f\left(j,\ell ,k\right)=\left\langle f,\psi_{j,\ell ,k}\right\rangle . \end{array}$$

Here the generator function *ψ* is compactly supported, with support contained in a rectangle whose length is controlled by the scaling parameter *j* ∈ ℕ, the orientation is controlled by ℓ ∈ ℤ and the location is controlled by *k* ∈ ℤ^2^. As we will show below in our numerical tests, when we computed the discrete Directional Ratio on segmented images of neuronal cultures, we observed very low values inside the neurites and much larger values (close to 1) inside the soma, according to the prediction of the theory [32]. However, near the boundary of the soma, the Directional Ratio is low, due to the strong directionality induced by the boundary. As a result, the application of a threshold allows us to identify a region *strictly inside the soma*, but not the entire soma. To detect the entire soma including the region close to the boundary, we need to ‘expand’ the region we discovered using the method of the Directional Ratio. To achieve this, we apply a classical level set method [33, 34].

**Fig 2:**
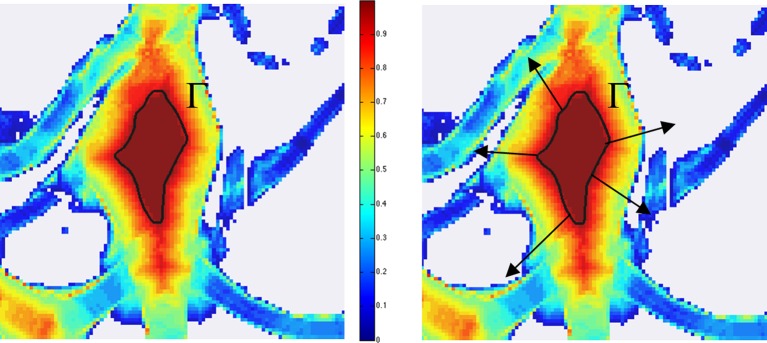
Application of level set method to detect soma area. The figure shows a detail from a segmented image of a neuron (MIP) where colors correspond to the values of the Directional Ratio values and range between 1 (=red) and 0 (=blue). The application of the threshold value 0.9 identifies a region strictly inside the soma, with boundary curve Γ (in the left panel). The level set method evolves the boundary curve Γ with a velocity in the normal direction (indicated by the arrows in the right panel) that depends on the magnitude of the gradient of the Directional Ratio.

Recall that the level set method is a variational approach introduced to track evolution of curves and shapes without having to parameterize these objects. The main idea is to identify a curve (or interface) Γ as the zero level set of a three-dimensional level set function *ϕ* and to follow the changes of Γ = {(*x*, *y*) : *ϕ*(*x*, *y*) = 0} from the evolution of *ϕ*. The motion of *ϕ* is determined by the *level set equation*

$$\begin{array}{c} \frac{\partial \phi }{\partial t}=v\left|\nabla \phi \right|, \end{array}$$

where *v* is the speed of propagation of Γ in the normal direction. In our numerical tests, we used the boundary curve of the region found by the Directional Ratio approach inside the soma as the initialization curve Γ of the level set evolution equation. We set the speed of propagation of Γ in the normal direction proportional to *M* − ∣∇(𝒟_*a*_ *f*)∣, where ∇(𝒟_*a*_ *f*) is the gradient of the Directional Ratio, pointing in the direction of the interior of the soma, and *M* is the maximum of the magnitude of the gradient of the Directional Ratio. This way, Γ evolves outwards, in the direction of the boundary of the segmented region (as shown in Fig. 2) and the velocity of evolution becomes slower and eventually stops when Γ reached the boundary of the segmented region. Note that, for our numerical implementation of the level set method, we have adapted the implementation of B. Sumengen [35] based on [33].

#### Separation of clustered somas

**Fig 3:**
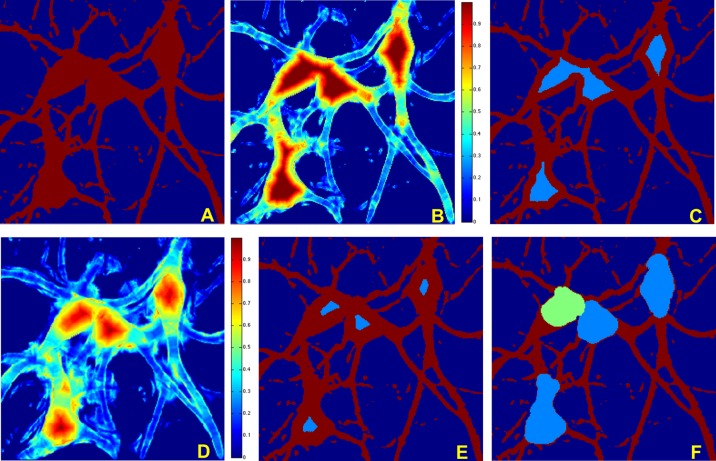
Separation of clustered somas. The figure illustrates the application of the multiscale Directional Ratio in combination with the level set method to separate contiguous somas on the MIP of a confocal image of a neuronoal network culture. (**A**) Segmented image (detail). (**B**) Directional Ratio plot using directional filters of length 20 pixels (note that the diameter of a soma is about 40 pixels). (**C**) The blue region shows the points where the Directional Ratio exceeds the threshold 0.9, identifying the more isotropic region. (**D**) Directional Ratio plot using directional filters of length 40 pixels; to note that the larger values of the Directional Ratio are now concentrated within a smaller set inside the blob-like regions. (**E**) The blue region shows the points where the Directional Ratio in panel D exceeds the threshold 0.9, identifying the more isotropic region; note that contiguous somas are now split into two regions. (**F**) Soma detection obtained from the application of the level set method, using the initialization curves determined by the boundary of the initial soma region in (**E**).

The method described above to separate somas from neurites in the segmented images of neuronal cultures may inadvertently detect multiple contiguous somas as a single soma. To address this issue, we designed a refinement of the soma extraction routine that proceeds as follows. After running our soma extraction routine, if the resulting soma area is too large (according to a criterion described below), we re-compute the Directional Ratio at a coarser scale, that is, by changing the scale parameter *j* in such a way that the supports of the analyzing functions are longer. By measuring the strength of the directional coherence at a coarser scale, the application of a threshold on the Directional Ration will produce some smaller regions contained in the inner part of the segmented area. This is illustrated in Fig. 3. Next, similar to the above procedure, we apply the level set method by using the boundary curves of these inner regions as the initialization curves of the level set evolution equation. As the numerical test below will show, by propagating these curves until they touch each other, we are able to separate contiguous somas.

We still need to clarify how to determine if the soma area detected by the algorithm is too big. This situation is interpreted as an indication that multiple somas appear in the MIP of the image as one contiguous soma region. In fact, we assume that somas in these images have similar surface areas (obviously, not similar shapes in general) and this observation applies to primary neuronal cultures which have fairly homogeneous populations. For instance, in our examples below, the vast majority of neurons in the cultures derive from pyramidal-like principal cells. To separate these large regions into multiple contiguous somas, we assume that soma areas are normally distributed. In practice, we applied the Kolmogorov-Smirnov test to validate this assumption on a representative set of confocal images of neuronal cultures and found that our assumption is correct. Based on this observation, we used the 3-*σ* rule as a criterion to identify regions containing multiple somas. That is, if the detected area differs from the expected soma area more than three times the estimated standard deviation, then we conclude that the area contains two somas. Similarly, for the case of multiples contiguous somas, we can argue that if a detected area differs from *N* times the expected soma area more than three times the standard deviation, then we conclude that the area contains *N*+1 somas. In practice, however, we found that it is extremely rare to find more than two contiguous somas in typical fluorescence images of neuronal cultures. Yet, as we observed, the method that we described can be applied to multiple contiguous somas.

Note that, in our implementation of the algorithm, the soma detection and separation of contiguous somas are processed automatically, without external intervention. Following the computation of Directional Ratio with filters of length 20 pixels (about 1/2 of a typical soma’s diameter), if the criterion indicated above signals the presence of areas containing two somas, then the Directional Ratio is re-computed using filter length of 40 pixels (about the diameter of a typical soma). This is illustrated in Fig. 3.

### 3D soma detection algorithm

In this section, we present an algorithm for the 3D detection of soma location and surface morphology from confocal image stacks of neuronal cultures. As indicated above, it is very challenging to extract volumetric information in this situation, due to the small number of pixels (typically about 15–30 pixels) available along the *z* direction and to the very low contrast of the images at the bottom of the stack. However, we present a method that is able to recover the surface morphology and capture the volume of the soma.

**Fig 4:**
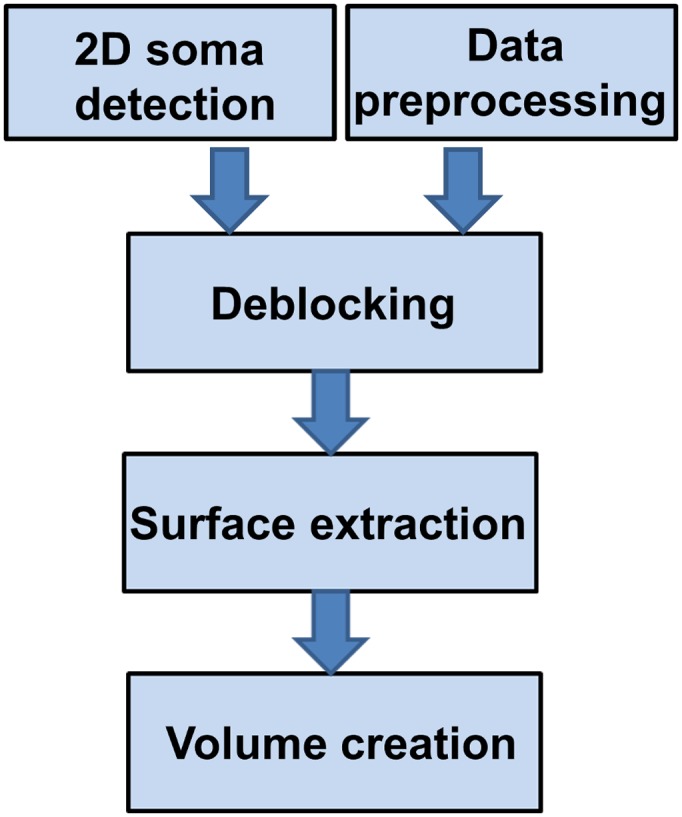
Proposed algorithm for 3D detection of soma location and surface morphology.

Our approach follows the procedure shown in Fig. 4 and it consists of the following steps: (1) a preprocessing routine to denoise the images and enhance the contrast of the images located at the bottom of the stack; (2) a 3D de-blocking routine that uses the 2D soma detection routine from above to extract subvolumes containing a single soma; (3) a surface detection routine based on the application the shearlet transform; (4) a routine that extracts the soma volume starting from the surface information. The sections below discuss each step in detail.

#### Data preprocessing

**Fig 5:**
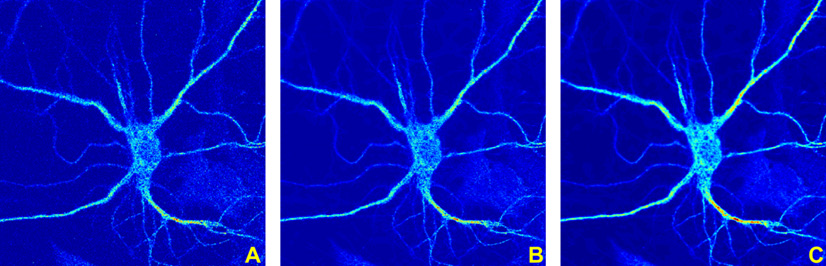
Image preprocessing. (**A**) MIP image of a representative neuron from a confocal image stack. (**B**) Shearlet-based denoised version of the same image. (**C**) Smoothed version of the denoised image, where the smoothing is obtained by convolving the image with a Gaussian kernel of size 3 × 3 and standard deviation *σ* = .6

Our preprocessing stage includes a denoising algorithm which is the same shealet-based algorithm described above for the 2D case and is applied to each image of the stack. However, we found that, even after denoising, the level of fluorescent intensity in the images was still rather uneven and this may cause some difficulties in the successive processing stages (e.g., holes in the extracted solid). To mitigate this effect, we applied a simple smoothing filter that is implemented by convolving the image stack with a 3*D* Gaussian kernel of size 3^3^ and standard deviation *σ* = .6. The value of *σ* and the size of the filter were determined after extensive numerical testing to provide the most satisfactory performance for surface detection. Fig. 5 illustrates our preprocessing method on a representative image of a neuron from a 3D confocal stack.

#### Deblocking

The 3D image stacks are deblocked into sub-stacks containing a single soma to reduce computational cost and simplify the processing needed to extract the surface morphology of the soma.

In confocal images of neuronal cultures, it is extremely rare to find completely overlapped somas. Thus, it is possible to partition the image plane into subregions containing a single soma. To deblock the 3D image stacks, we utilized the 2D soma detection algorithm presented above. From the output of this algorithm, we obtained information about the spatial localization of each soma from two-dimensional MIPs in directions parallel to each coordinate axis. We applied on these 2D MIPs the 2D-soma detection algorithm to derive masks *M*_*i*_ in the 2D-image plane (that we identify each time with the *xy*-plane) such that *M*_*i*_(*x*, *y*) = 1 if (*x*, *y*) belongs to the support of the *i*-th soma (in the image plane) and *M*_*i*_(*x*, *y*) = 0 otherwise. Finally, we obtained the 3D sub-stack containing the *i*-th soma as the sub-stack associated with the region of the image plane where *M*_*i*_ = 1. This non-necessarily rectangular mask tightly encloses each soma and contains the 2D-support region of the soma within each image slice.

#### Surface detection

The detection of the somas’ surface was achieved using a specialized shearlet-based surface detection routine that was developed by some of the authors [22, 23]. This routine was applied within the 3D subvolumes identified in the deblocking step.

**Fig 6:**
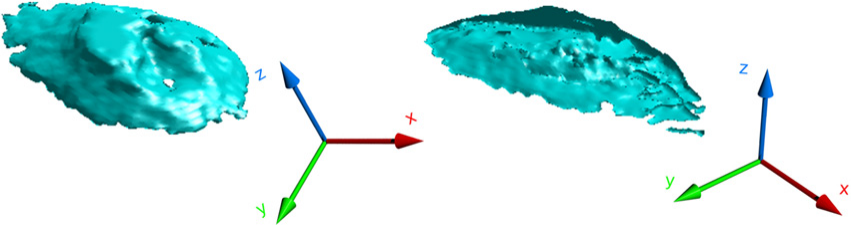
Shearlet-based surface detection of a soma. The figure shows two views of the surface morphology reconstruction of a soma obtained by processing a confocal image stack with the shearlet surface detection routine.

The advantage of applying the surface detection routine within these smaller volumes rather than over the entire stack is that the run-time of the algorithm is significantly reduced despite the linear computational cost growth with the increase of volume size. Fig. 6 illustrates the application of our surface detection routine for the extraction of the upper soma region from confocal image stacks of neuronal cultures. The figures show that this approach is very efficient at detecting the upper section of the soma’s surface. However, we found that, due to the very low image contrast in the bottom part of the stack, the algorithm is unable to extract a complete surface boundary in the bottom section of the soma, even though the mask we found above tightly encloses the soma.

#### Volume extraction

**Fig 7:**
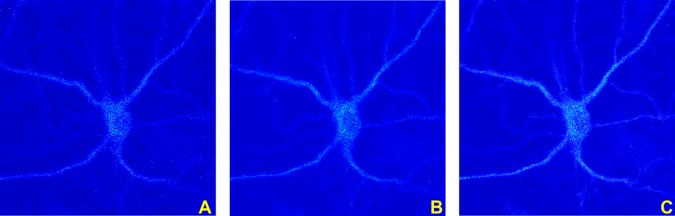
Bottom stack images. Representative images extracted from the bottom section of a confocal image stack of a neuronal culture. (**A**) Bottom image of the confocal stack; it is located the farthest from the light source. (**B-C**) Successive images in the stack, showing that the image contrast degrades significantly at the bottom of the confocal image stack.

Due to the difficulty in extracting the surface boundary of the soma in the bottom section of the confocal image stack, we cannot directly compute the volume of the soma from its surface information. Fig. 7 shows the bottom slices from a typical confocal image stack. Panels in this figure show that it is difficult to identify the soma’s contour.

**Fig 8:**
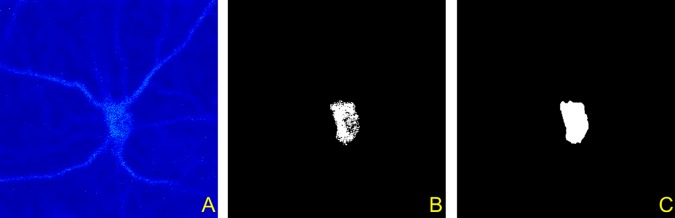
Extraction of soma support. The figure illustrates the detection of the cross section of the soma from a representative image taken from the bottom of a confocal image stack. (**A**) Denoised image. (**B**) Region obtained by applying the thresholding strategy within the region identified by the 2D mask as described in the text. (**C**) Detected support region obtained after applying a combination of filling and erosion operators to the image from Panel B.

Unfortunately, the application of a conventional image enhancement approach may also fail to improve the low signal-to-noise ratio of the images. On the other hand, the inspection of the images suggests that the soma’s support region does not change significantly from an optical section to the next one in this region of the stack (by contrast, there is often significant variability in the top optical sections). Therefore, we applied the following simple, yet effective strategy to detect a soma’s 3D support in each image stack. We computed the average of three successive images in a substack and use this value as an intensity threshold for the corresponding image. We applied this thresholding approach within the region identified by the mask we obtained from the 2D soma detection algorithm. We applied this approach only for the images located in the bottom half of the substack. Note that the extracted region may still contain some gaps. Hence, we applied a combination of filling and erosion operators [36, 37] to improve soma detection. An illustration of the application of this approach is given in Fig. 8 where we show a representative image of a neuron from the bottom of a confocal stack, the binary mask of the region obtained from the application of this localized thresholding strategy, and the final detected soma obtained after the application of the morphological operators. We remark that this simple thresholding procedure is successful because it is applied inside the box obtained by the masks *M*_*i*_ which tightly encases the soma.

**Fig 9:**
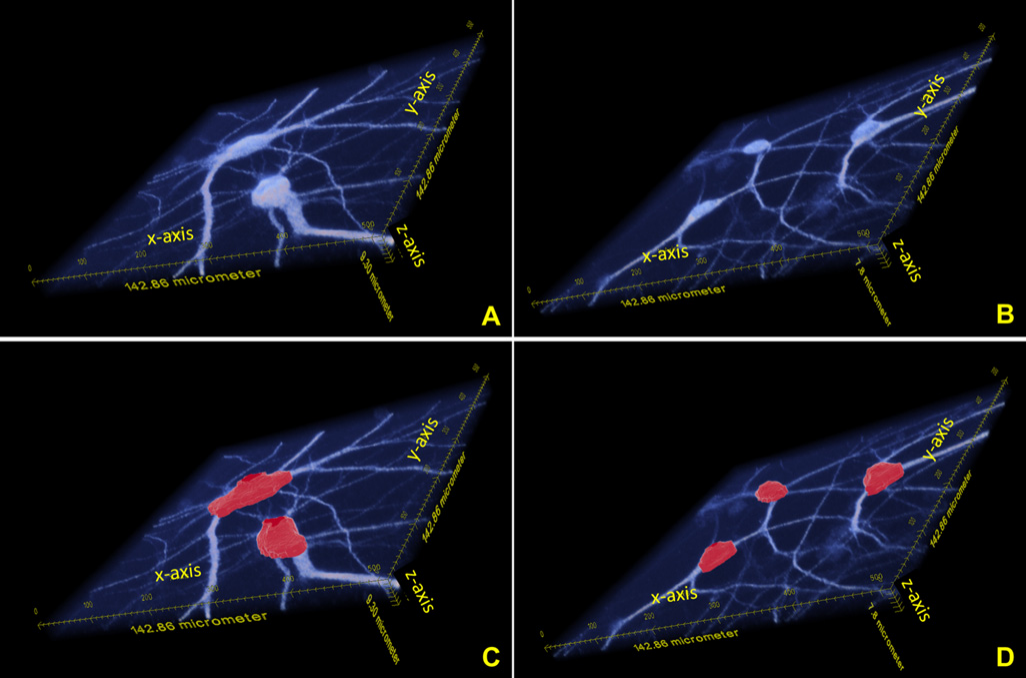
3D soma detection. (**A—B**) Visualization of two representative image stacks of neuronal cultures. Note that the stacks have a very limited extension along the *z* direction where less that 10 *μm* are available, corresponding to about 25–30 optical slices. (**C—D**) The images illustrate, in red color, the detection of the somas from the confocal image stacks shown above.

After having used the shearlet-based surface detection routine, we combined the regions the soma occupies in the images of the bottom half of the stack with the respective regions in the images of the top half of the stack to extract the entire volume. Fig. 9 shows some examples of soma extraction in 3D from confocal image stacks of neuronal cultures using the procedure described above. In each cases, the 3D image stacks consist of 512 × 512 × 25 voxels.

## Results

In this section, we illustrate the application of our soma detection algorithms on several confocal image stacks of neuronal cultures. For our tests, we considered several ‘standard’ field-of-view images in low-density neuronal cultures, as commonly used in phenotypic screenings of analytes for drug-discovery or biomarker identification (cf. [5, 6, 38, 39]). These images contain a relatively small number of neurons, typically about 5–10. We show below that our algorithm can automatically extract soma locations from any such image in about 40 seconds. Note that a large number of such images, usually 50–100 or more, need to be analyzed in studies like those in the cited literature. As we further explain below in the Discussion, our algorithms are scalable and can be applied without changes to larger images of neuronal cultures containing more neurons. To illustrate this capability, we also show the application of the soma detection algorithm on a tiled and stitched large field-of-view image containing more than 40 neurons.

### 2D soma detection

**Fig 10:**
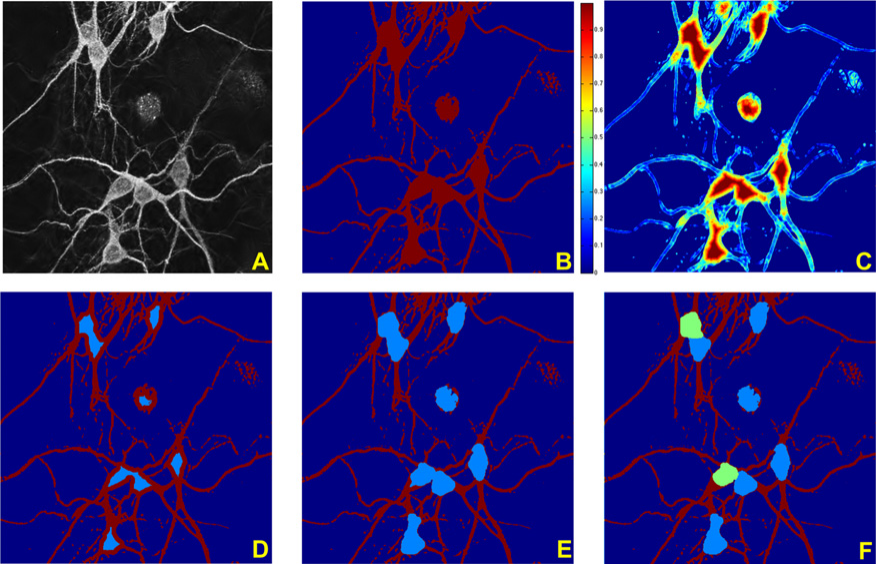
Illustration of 2D soma detection algorithm. (**A**) Denoised image, obtained using a shearlet-based denoising routine on the MIP of the image stack. Image size = 512 × 512 pixels (1 pixel = 0.28 × 0.28*μm*). (**B**) Segmented binary image. (**C**) Directional Ratio plot; the values range between 1, in red color (corresponding to more isotropic regions), and 0, in blue color (corresponding to more anisotripc regions); note that the Directional Ratio is only computed inside the segmented region, i.e., inside the red region in Panel B. (**D**) Detection of initial soma region, obtained by applying a threshold to the values of the Directional Ratio in Panel C. (**E**) Soma detection, obtained by applying the level set method with the initialization curve determined by the boundary of the initial soma region in Panel D. (**F**) Separation of contiguous somas; two regions from Panel E are recognized as too large and hence divided using the level set method.

We considered ten image stacks with different sizes to test our 2D algorithm for soma detection. The stacks we considered comprise between 10 and 25 images each and contain between 2 and 8 neurons. From each stack, we generated the MIP images and then processed the resulting 2D images using the algorithm described above. Fig. 10 illustrates the various steps of our algorithm on a representative MIP image from this set of ten image stacks. In particular, it shows the pre-processing, segmentation and soma detection on an images of size 512 × 512 pixels containing eight somas. The figure also illustrates the capability of the algorithm to separate contiguous somas, using the method of Directional ratio described above.

For the validation of the algorithm, we first tested the ability to identify the correct somas and separate the contiguous ones. The results are reported in Table 1 and show that the algorithm is able to correctly identify and separate the somas in all 10 confocal image stacks.

**Table 1:** Validation of soma count.

Stack	Number of somas	Correct detection	False detections	Contiguous Soma Presence
Stack 1	7	7	0	No
Stack 2	6	6	0	Yes
Stack 3	7	7	0	Yes
Stack 4	8	8	0	Yes
Stack 5	4	4	0	No
Stack 6	7	7	0	No
Stack 7	5	5	0	No
Stack 8	4	4	0	No
Stack 9	2	2	0	No
Stack 10	5	5	0	No

Performance on soma detection and separation of contiguous somas.

To quantitatively validate the performance of our algorithm on soma segmentation, we employed standard statistical measures of the performance of a binary classification test [40] whose definitions are as follows. The *True Positive Rate* *TPR* (also called *Sensitivity*) measures the proportion of correctly identified soma pixels with respect to the total number of true soma pixels, which are manually identified by a domain-expert (who identifies somas without knowledge of the algorithm results). That is, denoting by *TP* (= true positive) the number of correctly identified soma pixels and by *FN* (= false negative) the number of true soma pixels incorrectly rejected, we define:

$$\begin{array}{c} T\;P\;R=\frac{T\;P}{T\;P+F\;N}. \end{array}$$

The *False Positive Rate* *FPR* (which is the complement of the *Specificity*) measures the proportion of pixels incorrectly identified as soma pixels with respect to the total number of true soma pixels. That is, denoting by *FP* (= false positive) the pixels incorrectly selected as soma pixels, we adopted a special rate for the purposes of this work defined by

$$\begin{array}{c} F\;P\;R=\frac{F\;P}{T\;P+F\;N}. \end{array}$$

This rate is a penalty akin to wrong soma pixel detections. When our *FPR* is compared with the traditional *FPR* given by

$$\begin{array}{c} F\;P\;R=\frac{F\;P}{T\;N+F\;P}, \end{array}$$

one realizes that the commonly used *FPR* would be practically equal to zero because false soma detections are always in numbers that are an order of magnitude less than the number of background voxels, due to the inherent sparsity of the neuronal tissue in these images. Hence, we decided to introduce a new metric which expresses false soma detections as a percentage of soma volume measured in pixels/voxels. Finally, the *Dice Coefficient* *DC* (also called F1 score), that is used to compare the similarity between two samples or measures, is

$$\begin{array}{c} D\;C=\frac{2\;T\;P}{2\;T\;P+F\;N+F\;P}. \end{array}$$

Note that the denominator 2*TP* + *FN* + *FP* = *TP* + *FP* + *FN* + *TP* is the sum of the detected pixels and the true soma pixels.

**Fig 11:**
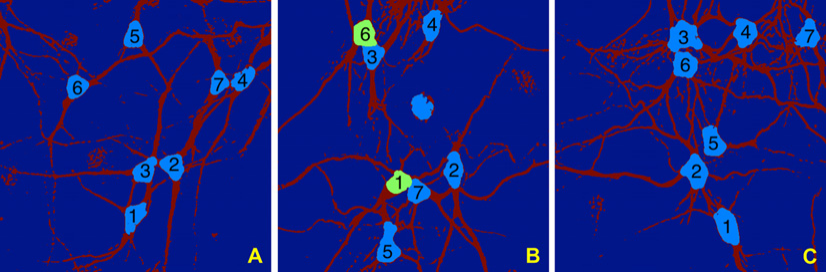
Performance of soma segmentation. 2D segmentation and soma detection of representative MIP images. The Performance metrics for the segmentation of the somas contained in these images are reported in Table 2.

The results, reported in Table 2 show the True Positive Rate, False Positive Rate and Dice Coefficient for the somas shown in Fig. 11. The results in the tables show that our method yields average *TPR* equal to 0.95, indicating that we get a very high proportion of true soma pixels; the value of the average *FPR* is 0.18, indicating that our approach tends to err on the side of false positives (i.e., we tend to over-segment). The average Dice coefficient is 0.89, indicating that the automated soma detection is very close to the manual segmentation overall.

**Table 2:** Segmentation performance.

Image and soma	*TPR*	*FPR*	*DC*
A 1	0.83	0.04	0.88
A 2	0.95	0.06	0.94
A 3	0.97	0.09	0.94
A 4	0.84	0.08	0.87
A 5	0.97	0.05	0.96
A 6	0.98	0.06	0.96
A 7	0.85	0.09	0.87
B 1	0.98	0.14	0.92
B 2	0.79	0.12	0.82
B 3	0.92	0.08	0.92
B 4	0.99	0.14	0.92
B 5	1	0.6	0.76
B 6	0.94	0.3	0.83
B 7	0.99	0.18	0.91
C 1	0.97	0.09	0.94
C 2	0.99	0.12	0.93
C 3	0.99	0.49	0.79
C 4	0.99	0.15	0.92
C 5	0.94	0.28	0.84
C 6	1	0.31	0.86
C 7	0.98	0.25	0.88
average	0.95	0.18	0.89

Performance metrics results on the soma segmentation of Fig. 11. Labels A, B and C refer to panels A, B and C, respectively, of Fig. 11. TPR = True Positive Rate, FPR = False Positive Rate; DC = Dice Coefficient.

**Fig 12:**
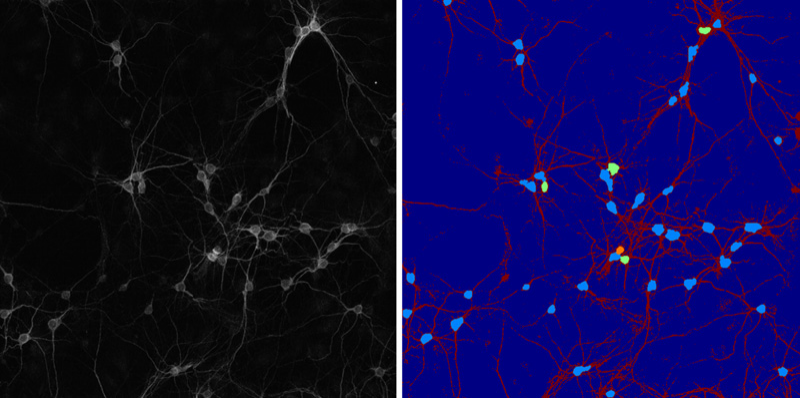
2D soma detection on large field-of-view image. Left: Tiled and stitched confocal fluorescent image (MIP view) of a neuronal culture. Image size = 1894 × 1894 pixels (1 pixel = 0.28 × 0.28*μm*). (**B**) Segmented binary image. Right: Soma detection and separation of contiguous somas. Segmented neurites are shown in red color. Detected somas are shown in light blue; in case of contiguous somas, the separated somas appear in green and orange colors.

To illustrate the capabilities of our approach on a larger field-of-view image, we applied our 2D soma detection algorithm on a tiled and stitched large field-of-view image of size 1894 × 1894 pixels containing about 45 neurons. The soma detection and segmentation result is reported in Fig. 12. The figure shows that our algorithm can reliably detect essentially all somas also in this larger image. Note that the soma detection algorithm is not expected to work for the somas overlapping the border of the image, as their shapes may be inconsistent with the model used by our method based on the Directional Ratio. Our method is also very effective in separating contiguous somas, even though it fails in some cases. About the center of the figure, three contiguous somas are correctly separated (shown in light blue, green and orange colors); however, in the top right region, where there is another set of three contiguous somas, the algorithm is only able to separate two of them. This is due to the fact two of the three soma are rather small and the area they occupy is not recognized as a location of multiple somas.

### 3D soma detection

We considered several image stacks with different sizes to test our 3D algorithm for soma detection. Some of these stacks are shown in Fig. 9. The image stacks were processed using the 3D algorithm presented above and some representative visual results are shown in the same Figure.

To validate the performance of our shearlet-based surface detection routine, we used a synthetic 3D image consisting of an ellipsoid and the Pratt’s figure of merit as objective measure of performance.

The *Pratt’s Figure of Merit* was originally introduced by Pratt to assess the performance of edge detectors [41] and is defined to penalize (i) the missing true edge/boundary points (false negatives), (ii) the points miss-classified as edge points due to noise (false positives) and (iii) the failure to detect the correct edge location. Given the detected edge pixels *N*_*I*_ and the true edge pixels *N*_*B*_, the Pratt’s Figure of Merit is defined as:

$$\begin{array}{c} FOM=\frac{1}{\max(N_{I},N_{B})}\sum_{i=1}^{N_{B}}\frac{1}{1+\alpha \;d_{i}^{2}} \end{array}$$

where *d*_*i*_ is the distance between a detected edge pixel and the nearest true edge pixel, and $\alpha =\frac{1}{9}$ is an empirical calibration constant. *FOM* varies in the range [0, 1], where 1 is the optimal value. Even though Pratt’s FOM was originally defined for edges in planar images, it can be adapted to assess the quality of surface detection. We tested the performance of our shearlet-based surface detection algorithm, assessed in terms of the Pratt’s FOM, on an ellipsoid that is convolved with a Gaussian smoothing kernel, for varying choices of the standard deviation *σ*. We found that the value of FOM is above 0.9, implying negligible loss in detection accuracy, as long as *σ* is below 0.6.

We recall that our algorithm for the extraction of the volume of the soma from the 3D confocal image stacks combines the information of the surface of the soma on the top half of the stack, with the information about the soma’s support in the bottom half of the stack, extracted with the localized thresholding strategy described above. Similar to the 2D case, we validated the performance of the detection of the soma volume using True Positive Rate, False Positive Rate and Dice Coefficient. Table 3 reports the performance metrics, in terms of the True Positive Rate, our special False Positive Rate and Dice Coefficient, for several somas extracted from the image stacks of Fig. 9. Note that stack 1 has dimensions 142.86*μm*(512) × 142.86*μm*(512) × 9.30*μm*(31) (in parenthesis is the pixel number). Similarly for stack 2: 142.86*μm*(512) × 142.86*μm*(512) × 7.80*μm*(26); for stack 3: 142.86*μm*(512) × 142.86*μm*(512) × 8.70*μm*(29); for stack 4: 142.86*μm*(512) × 142.86*μm*(512) × 9.00*μm*(30). As in the 2D case, the gold standard for the metrics used is the manual segmentation obtained by a domain expert, where the domain expert extracted the boundary of the soma in each optical slice in order to derive the volume. Note that the performance values are slightly lower that the 2D measures. This may be attributed to the difficulty in assessing the soma volume in the lowest optical slices, where the image contrast is very low; to the uncertainty associated to the slice-by-slice manual validation, and to the fact that surface detection is a natively 3D process eventually interpolating between neighbouring voxels in consecutive optical slices.

**Table 3:** 3D Segmentation performance.

Stack, Soma	*TPR*	*FPR*	*DC*
Stack 1, soma 1	0.86	0.06	0.90
Stack 1, soma 2	0.88	0.10	0.89
Stack 2, soma 1	0.79	.07	0.85
Stack 2, soma 2	0.83	0.10	0.86
Stack 2, soma 3	0.83	0.11	0.86
Stack 3, soma 1	0.78	0.05	0.85
Stack 4, soma 1	0.95	0.23	0.87
Average	0.85	0.10	0.87

Performance metrics results on 3D soma segmentation. TPR = True Positive Rate, FPR = False Positive Rate; DC = Dice Coefficient.

### Computational efficiency, hardware and software

We implemented the numerical codes for the method illustrated above using MATLAB 7.12.0 (R2011a). The tests for the 2D soma detection were performed using a MacBook with Intel Core i7 2.66 GHz and 4 GB RAM. Even though we did not optimize the computational efficiency of the routines, the computation times were very reasonable. On a 2D image of size 512 × 512 pixels, the average computational time for the shearlet-based denoising was approximately 11 seconds; the average computational time for the 2D segmentation routine was approximately 8 seconds; the computational time of the Directional Ratio routine that was used to initialize the soma detection depended slightly on the choice of parameters (number of directional bands and filter length) and ranged between 0.2 and 0.6 seconds; the average computational time for the level set method implementation and completion of soma segmentation was about 25 seconds. Tests for the 3D soma detection were performed using MATLAB 7.10.0.499(R2010a) on a 64 bit window 7 machine with Intel Core i5 2.50 GHz and 4 GB RAM. For the 3D soma detection, each image stack was first denoised slice-by-slice using the 2D Shearlet based routine indicated above. On an image stack of size 512 × 512 × 30, the average computational time for denoising was about 330 seconds, corresponding to an average of about 11 seconds for denoising each slice. The 3D soma surface extraction and volume construction took on average 155 seconds for a single soma. Note that this 3D routine calls the 2D-soma detection routine, thus the overall computational time is affected by the number of directional filters used in the implementation of the 3D shearlet transform. In our tests, we used 12 directional filters. Our Matlab routines for shearlet-based denoising and Directional Ratio are available at www.math.uh.edu/~dlabate/software.html. As mentioned above, we used the level set method implementation of Sumengen [35].

## Discussion

The methods we proposed for the automated detection of soma location and morphology in confocal images of neuronal cultures is scalable and can be easily applied to images larger than those considered in this paper, containing a larger number of cells. In fact, all steps of the algorithm, i.e., preprocessing, segmentation and soma extraction, can be performed without changes on larger images, provided that hardware can manage the data size (e.g., sufficient computer RAM). The computational cost of our algorithms depends linearly on the size of the data, which is reasonable for most applications. Several standard methods can be applied for speed improvement and management of larger data sets within the proposed framework.

The routines for denoising and surface detection, in particular, rely on the discrete shearlet transform. These routines are scalable and their computational cost grows linearly with the data size [23, 24]. Recently, a faster implementation of the discrete shearlet transform that uses a Graphic Processor Unit (GPU) was introduced and was reported to achieve a 20–30 times speed improvement over the Matlab implementation used for the results reported in this paper [42]. This implementation can be easily adapted to our algorithm since it implements the same shearlet decomposition and the code is publicly available [42].

The main computational cost of our algorithms is due to the routine for the soma segmentation that applies a level set method and requires the numerical solution of a partial differential equation. This routine is scalable and a GPU implementation was proposed [43] that could possibly be adapted to achieve computational speed improvement.

Parallelization is also a valuable strategy for handling larger images and reducing computational time of the algorithm. The deblocking strategy used in our method to separate the image stacks into sub-volumes makes it possible to parallelize our routines for volume and surface extraction. The level-set method can also be applied in parallel for each soma, following the application of the Directional Ratio routine. However, the Directional Ratio routine must be computed for each image in a stack, in order to identify soma locations and possible contiguous somas.

The accuracy of our algorithms critically depends on the segmentation routine since the soma area is found *within* the segmented region. Any pixels discarded during segmentation will not be classified as soma pixels. Despite this potential weakness, we found that the segmentation routine is very reliable for the type of data we consider, as clear the performance metric results showing consistently high values for the True Positive Rate. Consequently, we consistently identify pixels in neurites with high accuracy during segmentation. On the other hand, the performance metric tables show that, even with respect to the very penalizing FPR we adopted, we score reasonably low values of False Positive Rate. However, in some cases, this penalizing FPR tends to be relatively high indicating that, in general, we tend to positively identify a non-negligible number of pixels that are not in the soma. We attribute the main cause of this type of error to the level set method and, more precisely, the fact that the level curve propagated by the evolution equation does not always stop within the soma. For example in the detection of soma 5 in Fig. 11, Panel B, and soma 3 in Fig. 11, Panel C, we measure large values of FPR because the level set curve is propagated beyond the soma and partially inside the neurites. Note that, in these cases, the shape of the somas is rather elongated and the neurites emerging from the somas are not very thin, making it rather difficult to establish where the soma ends and the neurites start. Nevertheless, we think that it is possible to refine the algorithm in order to control this error in future work. For example, we could introduce a constraint or a penalty term in the level set routine to ensure that the detected soma region does not become too elongated.

Even though our algorithms were designed to process confocal images of neuronal cultures, several of our ideas are applicable to other types of imaging data. In particular, the segmentation routine has been tested on other confocal image stacks [28] and is expected to work on brightfield microscopy images as well. Similarly the method of Directional Ratio and surface detection based on the shearlet transform are expected to work with other types of imaging data. In addition, our shearlet-based surface detection routine can be considered as a useful alternative to Rayburst sampling methods used by other authors, such as [16]. With respect to the Rayburst sampling method, our approach is computationally less intensive as the shearlet transform requires a number of computation essentially linear with the data size. Moreover, it is less sensitive to local irregularities of a surface due to the intrinsic smoothing effect of shearlet filtering. On the other hand, our de-blocking routine is specifically designed for confocal images of neuronal cultures, where only a small number of pixels are available along the *z* axis and neurons don’t overlap along the *z* direction. For neuronal tissues, where it is possible to have higher numbers of optical slices, neurons may overlap in a truly 3D sense. In this latter case, we anticipate that de-blocking can be adapted to resolve contiguous cells in each of the three coordinate planes and thus separate in 3D contiguous cells.

## Conclusion

We have introduced a novel method for the automated detection of soma location and morphology in confocal images of neuronal cultures. In addition to the usual difficulties associated with processing fluorescent images, this task presents an additional challenge in that these types of confocal image stacks usually contain a small number of images (about 15–25). Consequently, only a small number of pixels are available along the *z*-direction and the use of standard 3D filters to process the data volume is inefficient or impractical. The approach we developed addresses these challenges by relying on a number of innovative ideas and techniques ranging from Fourier Analysis and differential equations, to statistics and computer vision. Accurate extraction of the somas in MIP images is achieved using an SVM-based segmentation routine followed by the application of Directional Ratio, a method derived from the theory of directional multiscale representations, and a level set method. This approach is also applied to reliably and efficiently separate contiguous somas in MIP images. Next, assisted by the 2D soma detection result, the accurate extraction of the surface and volume of the soma from the 3D image stack is obtained with an application of another multiscale method, the shearlet transform.

Automated detection and segmentation of somas in confocal images of neuronal cultures is a fundamental analytical tool not only for the identification and discrimination of neurons but more generally for the detection, analysis and profiling of complex phenotypes from cultured neuronal networks. The methods proposed and illustrated in this paper will facilitate the development of a high-throughput quantitative platform for the study of neuronal networks for applications including High Content analysis.

## References

[1] Lang P, Yeow K, Nichols A, Scheer A (2006) Cellular imaging in drug discovery. Nat Rev Drug Discov 5: 343–356. 10.1038/nrd2008 16582878

[2] Gough AH, Johnston PA (2007) Requirements, features, and performance of high content screening platforms. Methods Mol Biol 356: 41–61. 16988394 10.1385/1-59745-217-3:41

[3] Huang Y, Zhou X, Miao B, Lipinski M, Zhang Y, et al (2010) A computational framework for studying neuron morphology from in vitro high content neuron-based screening. J Neurosci Methods 190: 299–309. 10.1016/j.jneumeth.2010.05.012 20580743 PMC3184395

[4] Cornelissen F, Verstraelen P, Verbeke T, Pintelon I, Timmermans JP, et al (2013) Quantitation of chronic and acute treatment effects on neuronal network activity using image and signal analysis: toward a high-content assay. J Biomol Screen 18: 807–819. 10.1177/1087057113486518 23606652

[5] Shavkunov AS, Wildburger NC, Nenov MN, James TF, Buzhdygan TP, et al (2013) The fibroblast growth factor 14voltage-gated sodium channel complex is a new target of glycogen synthase kinase 3 (GSK3). J Biol Chem 288: 19370–85. 10.1074/jbc.M112.445924 23640885 PMC3707642

[6] Sharma K, Choi SY, Zhang Y, Nieland T, Long S, et al (2013) High-throughput genetic screen for synaptogenic factors: Identification of LRP6 as critical for excitatory synapse development. Cell Reports 5: 1330–1341. 10.1016/j.celrep.2013.11.008 24316074 PMC3924421

[7] Al-Kofahi KA, Lasek S, Szarowski DH, Pace CJ, Nagy G, et al (2002) Rapid automated threedimensional tracing of neurons from confocal image stacks. IEEE Trans Inf Technol Biomed 6:171–187. 10.1109/TITB.2002.1006304 12075671

[8] Al-Kofahi Y, Dowell-Mesfin N, Pace C, Shain W, N TJ, et al (2008) Improved detection of branching points in algorithms for automated neuron tracing from 3D confocal images. Cytometry A 73:36–43. 10.1002/cyto.a.20499 18067123

[9] Svoboda K (2011) The past, present, and future of single neuron reconstruction. Neuroinformatics 9: 87–98. 10.1007/s12021-011-9097-y 21279476

[10] Pawley JB (2006) Handbook of biological confocal microscopy. New York (N.Y.): Springer.

[11] Weaver CM, Pinezich JD, Lindquist WB, Vazquez ME (2003) An algorithm for neurite outgrowth reconstruction. J Neurosci Methods 124: 197–205. 10.1016/S0165-0270(03)00017-7 12706850

[12] El-Laithy K, Knorr M, Ks J, Bogdan M (2012) Digital detection and analysis of branching and cell contacts in neural cell cultures. J Neurosci Methods 210: 206–219. 10.1016/j.jneumeth.2012.07.007 22841629

[13] He W, Hamilton TA, Cohen AR, Holmes TJ, Pace C, et al (2003) Automated three-dimensional tracing of neurons in confocal and brightfield images. Microsc Microanal 9: 296–310. 10.1017/S143192760303040X 12901764

[14] Vallotton P, Lagerstrom R, Sun C, Buckley M, Wang D, et al (2007) Automated Analysis of Neurite Branching in Cultured Cortical Neurons Using HCA-Vision. Cytom Part A 71: 889–895. 10.1002/cyto.a.20462 17868085

[15] Al-Kofahi Y, Lassoued W, Lee W, Roysam B (2010) Improved automatic detection and segmentation of cell nuclei in histopathology images. IEEE Trans Biomed Eng 57: 841–852 10.1109/TBME.2009.2035102 . 19884070

[16] Yan C, Li A, Zhang B, Ding W, Luo Q, et al (2013) Automated and accurate detection of soma location and surface morphologyin large-scale 3D neuron images. PLoS ONE 8: 1–12. 10.1371/journal.pone.0062579 PMC3634810 23638117

[17] Rodriguez A, Ehlenberger D, Hof P, Wearne S (2006) Rayburst sampling, an algorithm for automated three-dimensional shape analysis from laser scanning microscopy images. Nature protocols 1: 2152–2161. 10.1038/nprot.2006.313 17487207

[18] Kutyniok G, Labate D (2012) Shearlets: Multiscale Analysis for Multivariate Data. Springer.

[19] Guo K, Labate D (2009) Characterization and analysis of edges using the continuous shearlet transform. SIAM J Imaging Sci 2: 959–986. 10.1137/080741537

[20] Guo K, Labate D (2010) Analysis and detection of surface discontinuities using the 3D continuous shearlet transform. Appl Comput Harmon Anal 30: 231–242. 10.1016/j.acha.2010.08.004

[21] Guo K, Labate D (2012) Characterization of piecewise smooth surfaces using the 3D continuous shearlet transform. J Fourier Anal Appl 18: 488–516. 10.1007/s00041-011-9209-y

[22] Schug DA, Easley GR, OLeary DP (2011) Three-dimensional shearlet edge analysis. Proc SPIE 8058, Independent Component Analyses, Wavelets, Neural Networks, Biosystems, and Nanoengineering IX.

[23] Yi S, Labate D, Easley GR, Krim H (2009) A shearlet approach to edge analysis and detection. IEEE Trans Image Process 18: 929–941. 10.1109/TIP.2009.2013082 19336304

[24] Easley GR, Labate D, Lim W (2008) Sparse directional image representations using the discrete shearlet transform. Appl Numer Harmon Anal 25: 25–46. 10.1016/j.acha.2007.09.003

[25] Negi PS, Labate D (2012) 3-d discrete shearlet transform and video processing. IEEE Trans Image Process 21: 2944–2954. 10.1109/TIP.2012.2183883 22249714

[26] Guo K, Labate D (2007) Optimally sparse multidimensional representation using shearlets. SIAM J Math Anal 39: 298–318. 10.1137/060649781

[27] Easley GR, Labate D (2012) Image processing using shearlets In: Shearlets: Multiscale Analysis for Multivariate Data, Boston, MA: Birkhäuser Boston, Appl. Numer. Harmon. Anal pp. 283–325.

[28] Jimenez D, Papadakis M, Labate D, Kakadiaris I (2013) Improved automatic centerline tracing for dendritic structures In: Biomedical Imaging (ISBI), 2013 IEEE 10th International Symposium on. pp. 1050–1053.

[29] Jimenez D, Labate D, Kakadiaris IA, Papadakis M (2014) Improved automatic centerline tracing for dendritic and axonal structures Neuroinformatics: 1–18. 10.1007/s12021-014-9256-z 25433514

[30] Jimenez D, Labate D, Papadakis M (2015) Directional analysis of 3d tubular structures via isotropic well-localized atoms. Appl Comput Harmon Anal: 1–18.

[31] Labate D, Laezza F, Negi P, Ozcan B, Papadakis M (2014) Efficient processing of fluorescence images using directional multiscale representations. Math Model Nat Phenom 9: 177–193. 10.1051/mmnp/20149512 28804225 PMC5553129

[32] Ozcan B, Labate D, Jimenez D, Papadakis M (2013) Directional and non-directional representations for the characterization of neuronal morphology In: Wavelets XV (San Diego, CA, 2013), SPIE Proc. volume 8858, pp. 1050–1053.

[33] Osher S, Fedkiw RP (2003) Level set methods and dynamic implicit surfaces Applied mathematical science. New York, N.Y.: Springer.

[34] Sethian JA (1999) Level Set Methods and Fast Marching Methods. Cambridge University Press, 2 edition.

[35] Sumengen B (2005) Vision Research Lab at UC Santa Barbara. http://barissumengen.com/level_set_methods/index.html.

[36] Soille P (2003) Morphological Image Analysis: Principles and Applications. Secaucus, NJ, USA: Springer-Verlag New York, Inc., 2 edition.

[37] Gonzalez RC, Woods RE, Eddins SL (2003) Digital Image Processing Using MATLAB. Upper Saddle River, NJ, USA: Prentice-Hall, Inc.

[38] Dolmetsch R, Geschwind D (2011) The human brain in a dish: The promise of ipsc-derived neurons. Cell 145: 831–834 10.1016/j.cell.2011.05.034 . 21663789 PMC3691069

[39] Nieland TJF, Logan DJ, Saulnier J, Lam D, Johnson C, et al (2014) High content image analysis identifies novel regulators of synaptogenesis in a high-throughput rnai screen of primary neurons. PLoS ONE 9: e91744 10.1371/journal.pone.0091744 24633176 PMC3954765

[40] Altman DG, Bland JM (1994) Diagnostic tests. 1: Sensitivity and specificity. BMJ 308: 15–52. 10.1136/bmj.308.6943.1552 8019315 PMC2540489

[41] Abdou IA, W P (1979) Quantitative design and evaluation of enhancement/thresholding edge detectors. Proc IEEE 67(5): 753–766. 10.1109/PROC.1979.11325

[42] Gibert X, Patel V, Labate D, Chellappa R (2014) Discrete shearlet transform on GPU with applications in anomaly detection and denoising. EURASIP J Adv Sig Pr 2014: 64.

[43] Cates JE, Lefohn AE, Whitaker RT (2004) GIST: An interactive GPU-based level-set segmentation tool for 3D medical images. Med Image Anal 8: 217–231. 10.1016/j.media.2004.06.022 15450217

